# Alteration of m^6^A-Tagged RNA Profiles in Bone Originated from Periprosthetic Joint Infection

**DOI:** 10.3390/jcm12082863

**Published:** 2023-04-14

**Authors:** Yuanqing Cai, Xiaoqing Chen, Changyu Huang, Yang Chen, Chaofan Zhang, Zida Huang, Wenming Zhang, Yusen Tang, Xinyu Fang

**Affiliations:** 1 Department of Orthopaedics, National Regional Medical Center, Binhai Campus of the First Affiliated Hospital, Fujian Medical University, Fuzhou 350212, China; 2 Department of Orthopaedics, The First Affiliated Hospital of Fujian Medical University, Fuzhou 350005, China; 3 Department of Orthopaedics, The Second Affiliated Hospital of Xi’an Jiaotong University, Xi’an 710004, China; 4 Department of Orthopaedics, The 909th Hospital, School of Medicine, Xiamen University, Zhangzhou 363000, China; 5 Department of Orthopaedics, Quanzhou First Hospital Affiliated to Fujian Medical University, Quanzhou 362000, China

**Keywords:** N^6^-methyladenine, *Staphylococcus aureus*, periprosthetic joint infection, mRNA, profiles

## Abstract

Periprosthetic joint infection (PJI) is a devastating complication. This study aimed to unravel the veil of the N^6^-methyladenine (m^6^A) modification in PJI. Synovium, synovial fluid, sonication fluid and bone samples were collected intraoperatively from *Staphylococcus aureus* PJI and aseptic failure (AF) patients. The overall m^6^A level was detected by the m^6^A RNA methylation quantification kit, and the expression of m^6^A-related genes was quantified by real-time PCR and Western blot. Finally, an epitranscriptomic microarray and bioinformatics analysis were performed. We showed that there was a significant difference in overall m^6^A level between the PJI group and the AF group (PJI group had a higher overall m^6^A level). The expression level of METTL3 was higher in the PJI group than that in the AF group. There were 2802 differential m^6^A-modified mRNAs. Kyoto Encyclopedia of Genes and Genomes (KEGG) analysis showed that differential m^6^A-modified mRNAs were significantly enriched in the NOD-like receptor signaling pathway, Th17 cell differentiation and the IL-17 signaling pathway, which indicates that the m^6^A modification might be involved in the processes of infection and immune response, bone metabolism and programmed cell death in PJI. In summary, the present work demonstrated that m^6^A modification plays a role in PJI and might be a therapeutic target for developing effective treatment strategies.

## 1. Introduction

Periprosthetic joint infection (PJI) is a devastating complication after joint arthroplasties [1], which results in multiple revision surgeries and extensive antibiotic treatment [2]. Currently, the protocols for PJI treatment include one- or two-stage revision combined with administration of antibiotics for an extensive period. However, although revision surgeries were performed and antibiotics were administered, the incidence of PJI after revision is 4–17% [3]. Thus, sufficiently understanding the physiological and pathological mechanisms of PJI is of great importance to develop effective treatment strategies. *Staphylococcus aureus* (*S. aureus*), a common pathogenic bacteria of PJI [4], can employ many strategies to resist neutrophil-mediated killing and evade host immunity, and it leads to chronic or recurrent infection that is difficult to treat [5,6]. Recently, immunotherapy of PJI has got attention increasingly [6]. However, the pathological mechanisms in PJI have not yet been fully elucidated.

In recent years, various chemical modifications of RNAs have been reported. Among these, N^6^-methyladenine (m^6^A) is the most common post-transcriptional modification of eukaryotic mRNA, accounting for 80% of RNA methylation [7]. It is known that in most eukaryotes, methylation in the mRNA 5′UTR region plays an important role in mRNA splicing, stability, degradation, and so on, while methylation in the 3′UTR region contributes to mRNA nuclear transport, translation initiation and maintenance of mRNA structural stability together with polyA-binding proteins [8]. The m^6^A methylation modification has been proven to be reversible, involving the methyltransferase complex (namely writers) that mainly includes methyltransferase like (METTL)3, METTL14 and wilms tumor 1-associating protein (WATP); demethylases (namely erasers), including AlkB homology 5 (ALKBH5) and fat mass and obesity associated protein (FTO); and m^6^A-binding proteins (namely readers) including YTHDF and others [9,10]. The m^6^A methylation modification has been demonstrated to be widely involved in mammalian development, immunity, tumor formation and metastasis, stem cell renewal, fat differentiation and other life processes [7,11,12,13,14].

Several recent studies focused on the role of m^6^A modification in infection. Lichinchi G et al. [15] demonstrated that HIV-1 infection of CD4^+^ T cells caused the overall level of m^6^A modifications on viral and T cell RNA to increase significantly, and m^6^A-tagged mRNAs in CD4^+^ T cells are essential for viral gene replication. Furthermore, they showed that the expression of METTL3, METTL14 and ALKBH5 in CD4^+^ T cells can directly affect HIV-1 replication. Feng Z et al. [16] also showed that the overall level of m^6^A modification and METTLE3 were elevated in human dental pulp cells (HDPCs) induced by lipopolysaccharide (LPS) from bacteria. Additionally, METTL3 was involved in the regulation of the inflammatory response of HDPCs via regulating alternative splicing of MyD88.

However, the pathological and physiological roles of m^6^A modification in PJI are elusive. Therefore, the objective of this study was to unravel the mysterious veil of m^6^A methylation modification in PJI. To the best of our knowledge, this is the first study to investigate the relationship between m^6^A modification and PJI. This study might also provide a potential therapeutic target for PJI.

## 2. Materials and Methods

### 2.1. Patient Selection and Sample Collection

From January 2018 to December 2019, patients who underwent revision surgeries due to periprosthetic joint infection or aseptic failure (AF) in our center were enrolled. Periprosthetic tissues, synovial fluid, bone and sonication fluid were collected intraoperatively. Part of the periprosthetic tissues, synovial fluid and sonication fluid underwent microbial culture for pathogenic bacteria detection, and the remaining portion as well as the bone were stored at −80 °C for further experiments. A PJI diagnosis was made according to Musculoskeletal Infection Society (MSIS) criteria for PJI [17]; one of the following must be met for diagnosis of PJI: (1) a sinus tract communicating with the prosthesis; (2) a pathogen is isolated by culture from two separate tissue or fluid samples obtained from the affected periprosthetic joint; (3) four of the following six criteria exist: (a) elevated erythrocyte sedimentation rate (ESR) and C-reactive protein (CRP); (b) elevated synovial fluid WBC counts; (c) elevated synovial fluid neutrophil percentage; (d) presence of purulence in the affected joint; (e) isolation of a microorganism in one periprosthetic tissue or synovial fluid culture and (f) >5 neutrophils per high-powered field in 5 high-power fields observed from histologic analysis of periprosthetic tissue at ×400 magnification. 

For an epitranscriptomic microarray analysis of m^6^A-modified mRNA and lncRNA, bone samples from PJI cases caused by *S. aureus* were selected, and samples from AF cases matched by sex, age, body mass index (BMI), concomitant disease etc. were used as controls.

### 2.2. Hematoxylin–Eosin (HE) Staining 

Synovium was removed intraoperatively and immersed in 10% formalin. After that, synovium was dehydrated under serial alcohol gradient from low to high concentrations and underwent wax immersion and embedding. The wax blocks were fixed on a slicer and sliced into thin slices. The slices were successively put into xylene Ⅰ (20 min), xylene Ⅱ (20 min), anhydrous ethanol Ⅰ (10 min), anhydrous ethanol Ⅱ (10 min), 95% alcohol (5 min), 90% alcohol (5 min), 80% alcohol (5 min) and 70% alcohol (5 min). After washing with distilled water, slices were stained with hematoxylin for 10 min, rinsed with water, differentiated with hydrochloric ethanol and stained with eosin for 1 min. Then they underwent microscope examination. The experiment was performed in triplicate and was performed in a pathology lab (37 °C).

### 2.3. Quatification of the Overall Level of m^6^A Methylation Modification

Total RNA of bone collected intraoperatively was isolated by the RNA extraction kit RNAiso Plus (Takara Bio Inc., Japan) according to manufacturer’s instructions. Briefly, 15 mg of bone tissue was ground to a powder with 2 mL of liquid nitrogen, and then RNAiso was added. After transferring to a 1.5 mL Eppendorf (EP) tube, 0.2 mL of chloroform was added into each tube, and it was shaken violently up and down for 15 s and left to stand for 5 min. They were then centrifuged at 12,000× *g*, 4 °C for 15 min. The supernatants were mixed with an equal volume of isopropanol and centrifuged at 12,000× *g*, 4 °C for 10 min. After that, precipitation was dissolved in 75% ethanol and underwent centrifugation at 12,000× *g*, 4 °C for 5 min. Finally, ethanol was discarded, and DEPC was added to dissolve precipitation. Total RNA was stored at −80 °C. The overall level of m^6^A methylation was determined via EpiQuik ™ m^6^A RNA Methylation Quantification Kit (P-9005, Epigentek, Farmingdale, NY, USA). To begin with, a standard curve was generated with a positive control (PC, m^6^A oligos and was normalized to have 100% of m^6^A) and negative control (an RNA containing no m^6^A). The process included three main parts: (1) RNA binding: 2 μL of negative control (NC), 2 μL of diluted PC and 200 ng of bone RNA were added into strip wells with binding solution. (2) m^6^A RNA capture: diluted capture antibody, detection antibody and enhancer solution were added into wells in turn. (3) Signal detection: after incubation with developer solution, stop solution was prepared and added to stop the enzyme reaction. Finally, the absorbance was read on a microplate reader at 450 nm. This experiment was performed in triplicate.

### 2.4. Quantification of m^6^A-Related Gene Expression by qPCR

Total RNA was extracted RNAiso Plus (Takara Bio Inc., Japan) and reverse-transcribed into cDNA with a Symantec Reverse Transcription Kit (Thermo Fisher Scientific, NY, USA). Quantitative polymerase chain reaction (qPCR) was performed with a PCR instrument (Applied Biosystems, Shanghai, China), and TBGREEN (Takara, Japan) was used for fluorescence measurement. GAPDH was used as an internal control, and the primer sequences (Sangon Biotech, Shanghai, China) are shown in Supplementary File S1. The experiment was performed in triplicate.

### 2.5. Western Blot 

Bone was weighed and transferred into a mortar filled with liquid nitrogen, and the bone tissue was ground into powder. The powder was collected in a 1.5 mL EP tube, a lysis solution was added (200 μL lysate per 100 mg of bone tissue), the bone tissues were lysed on ice for 30 min, and the lysate was sucked from the EP tube into another EP tube. It was then centrifuged at 12,000 rpm at 4 °C and stored at −80 °C. The proteins were separated by 10% SDS-PAGE gels, and the separated proteins after electrophoresis were transferred to polyvinylidene fluoride membranes and blocked with 5% defatted milk powder for 2 h. Then the primary antibody anti-METTL3 (ab195352; Abcam; USA) and anti-GAPDH (AF7021; Affinity Biosciences, Cincinnati, OH, USA) was added and incubated overnight at 4 °C. Afterwards, the secondary antibody was added and incubated at room temperature for 2 h. After washing with TBST three times, the membranes were quantified using the ECL detection reagent. All experiments were performed in triplicate.

### 2.6. m^6^A-Tagged RNA Immunoprecipitation, Microarray Hybridization and Data Analysis

Total RNA from each sample was quantified using the NanoDrop ND-1000. Briefly, the total RNA was immunoprecipitated with anti-N6-methyadenosine (m^6^A) antibody. The modified RNAs were eluted from the immunoprecipitated magnetic beads as the “IP”. The unmodified RNAs were recovered from the supernatant as “Sup”. The “IP” and “Sup” RNAs were labeled with Cy5 and Cy3, respectively, as cRNAs in separate reactions using Arraystar Super RNA Labeling Kit. The cRNAs were combined together and hybridized onto an Arraystar Human mRNA and lncRNA Epitranscriptomic Microarray. After washing the slides, the arrays were scanned in two-color channels by an Agilent Scanner G2505C. Differentially m^6^A-methylated RNAs between two comparison groups were identified by filtering with log2 fold change (FC) > 1.5 and statistical significance (*p* < 0.05) thresholds. Hierarchical clustering and principal component analysis (PCA) were performed to show the distinguishable m^6^A-methylation pattern among samples.

Microararray data analysis was performed using Agilent Feature Extraction software. Raw intensities of immunoprecipitated RNAs were normalized and log2 transformed. The GO (gene ontology) analysis and pathway analysis on differentially m^6^A-tagged RNAs were perform via the GO (http://www.geneontology.org (accessed on 31 August 2021)) database and Kyoto Encyclopedia of Genes and Genomes (KEGG) database, respectively.

### 2.7. Statistical Analysis

All data were expressed as the means ± SD (standard deviation). Student’s *t* tests were used to compare the differences between two groups, it was considered that the difference was statistically significant when *p* ≤ 0.05. All statistical analysis was performed on GraphPad Prism Version 7.00.

## 3. Results

### 3.1. PJI Patients

**Figure 1:**
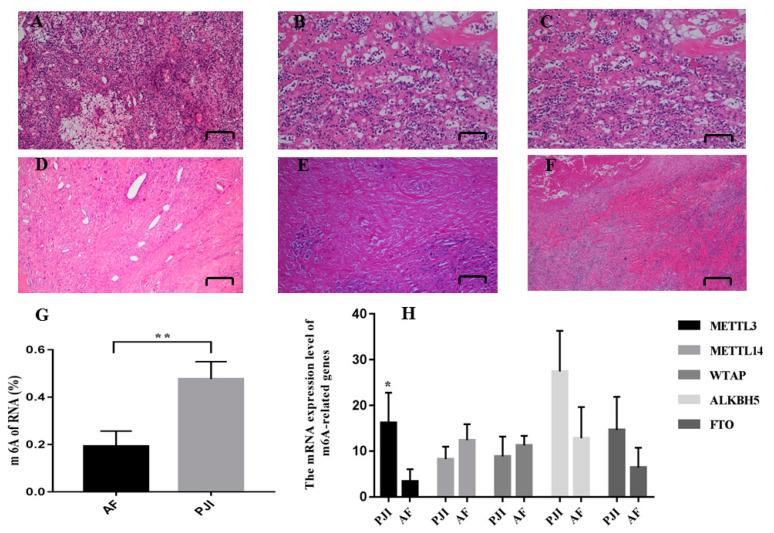
HE staining (**A**–**F**): inflammatory hyperplasia was observed in synovium of PJI, with inflammatory cells, plasma cell infiltration, and multinucleated giant cell reaction. The neutrophil counts > 50/HPF (high power filed). (**A**–**C**): PJI; (**D**–**F**): AF. The overall m6A level of PJI was higher than AF (**G**). The relative mRNA expression level of methylases and demethylases (**H**). * *p* < 0.05; ** *p* < 0.01.

A diagnosis of PJI was made according to the MSIS criteria. Synovium, synovial fluid and sonication fluid collected intraoperatively underwent microbial culture, and the synovium also underwent pathological detection. The culture results were judged by at least two microbiologists. Bone samples collected intraoperatively from PJI patients whose pathogenic bacteria was *S. aureus* were used in this study. Demographic characteristics of patients whose bone samples were used for Arraystar Human mRNA and lncRNA Epitranscriptomic Microarray analysis are listed in Supplementary File S2. Patients included in the PJI and AF group were selected and strictly matched, and patients with obesity and other comorbidities (assessed by Charlson comorbidity index) were excluded to minimize the impact of other factors. There were no differences in age, sex, BMI or Charlson comorbidity index between two groups. HE staining showed that there was inflammatory hyperplasia in synovium from PJI patients, with inflammatory cells, plasma cell infiltration, multinucleated giant cell reaction and neutrophil counts > 50/HPF (Figure 1A–F). 

### 3.2. The Overall m^6^A Level of PJI Group Was Significantly Higher Than That of AF Group

To compare the difference in overall m^6^A level between the PJI group and AF group, an m^6^A RNA methylation quantification kit was used to analyze RNAs extracted from bone samples by ELISA. We showed that the overall level of m^6^A modification in PJI group was significantly higher than that in AF group (Figure 1G), which clarified our previous hypothesis that m^6^A modification is involved in the pathophysiological process of PJI.

### 3.3. The Expression of RNA Methylase METTL3 Was Significantly Upregulated in PJI Group

**Figure 2:**
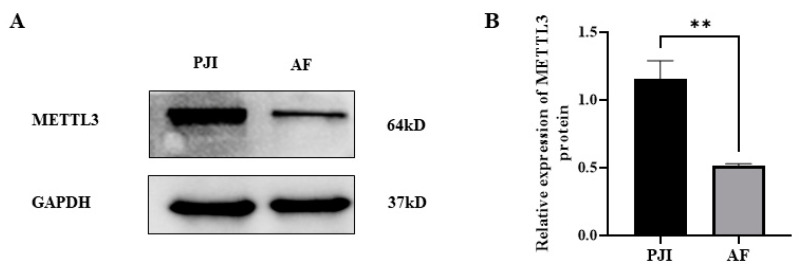
The expression of METTL3 in PJI and AF detected by Western blot (**A**) and statistical analysis (**B**). PJI: periprosthetic joint infection; AF: aseptic failure; ** *p* < 0.01.

In order to study the role of m^6^A-related genes in this process, real-time quantitative PCR was performed to detect the expression level of m^6^A-related genes. The results showed that the expression level of METTL3 increased, while there were not alternations in the expression of demethylase (ALKHB5 and FTO) (Figure 1H). Furthermore, the expression of METTL3 was also detected at the protein level by Western blot, and the results showed that the expression of METTL3 in the PJI group was higher than that in AF group (Figure 2). 

### 3.4. Alternations of m^6^A-Modified RNA Profiles in Bone Samples from PJI

**Figure 3:**
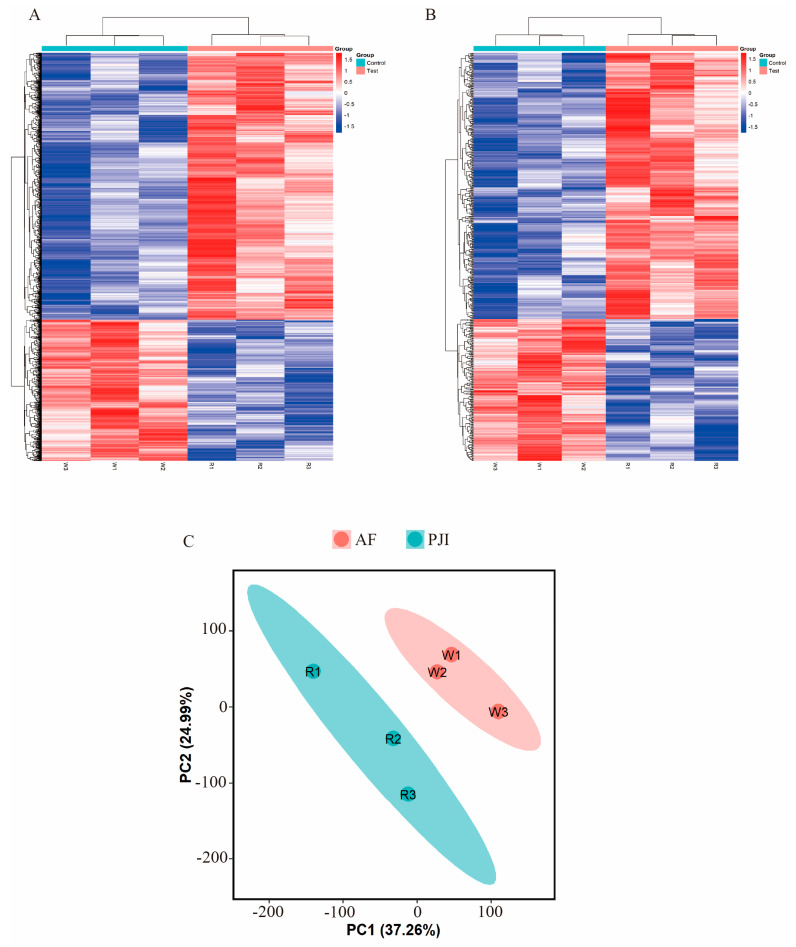
Hierarchical clustering analysis of differential m6A-modified mRNAs (**A**) and lncRNAs (**B**). Principal component analysis of differential m6A-modified mRNAs (**C**).

**Figure 4:**
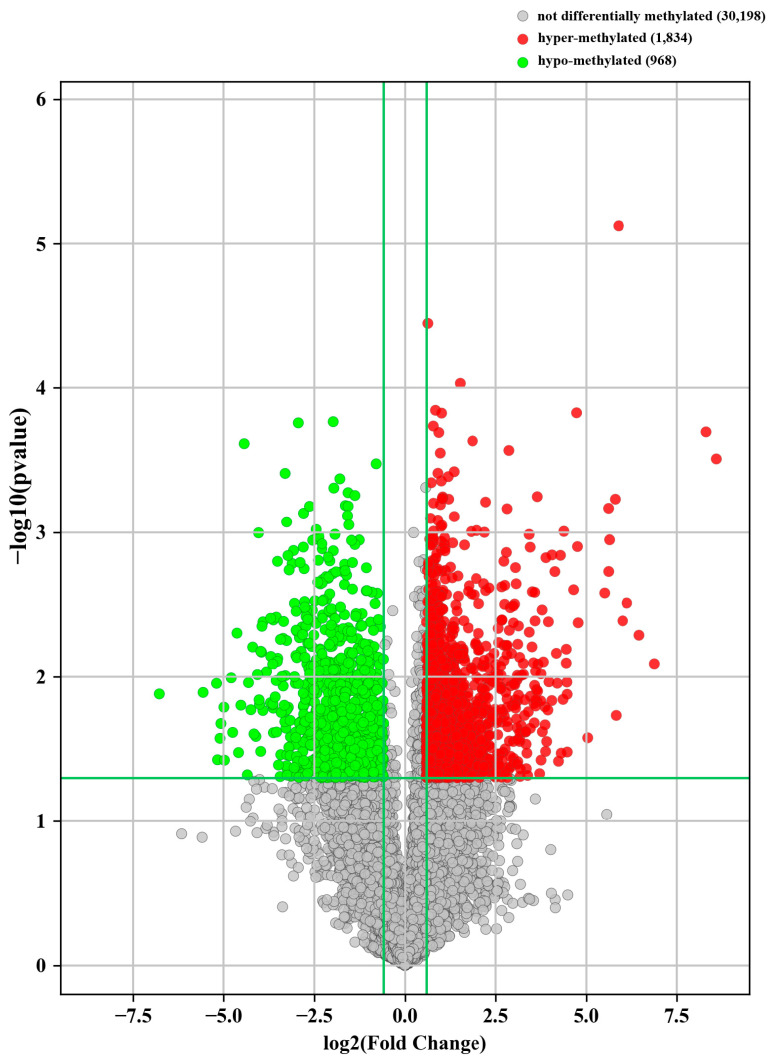
Volcano plot showed there were 2802 differentially methylated mRNAs (FC > 1.5, *p* < 0.05; with 1834 hypermethylated mRNAs and 968 hypomethylated mRNAs).

We have shown that the overall m^6^A level in PJI that was caused by *S. aureus* was significantly increased. To further study m^6^A-modified RNA profiles, we analyzed the whole genome map of m^6^A-modified RNA. The analysis results showed that there was a significant difference in m^6^A modification RNA profiles between the PJI group and AF group (Figure 3). There were 2802 differential m^6^A-modified mRNAs (1834 hypermethylated and 968 hypomethylated) (Figure 4). The top 20 mRNAs with differential m^6^A modification are listed in Supplementary File S3.

### 3.5. GO and KEGG Pathway Analysis of Differential m^6^A-Tagged mRNAs

**Figure 5:**
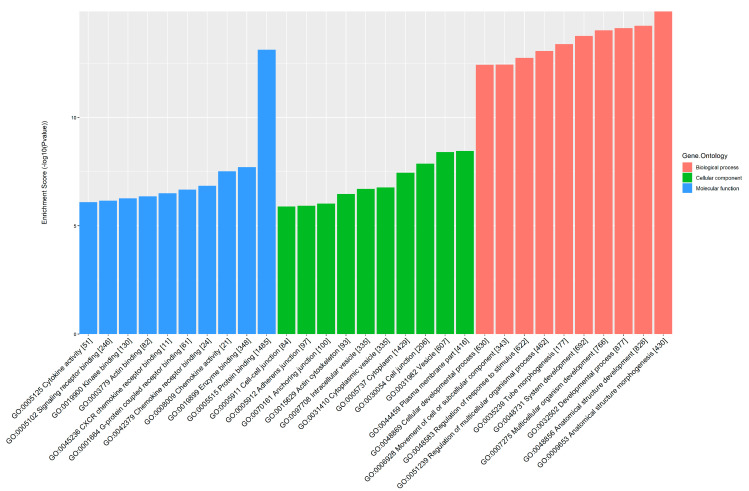
Gene ontology (GO) analysis of differential m6A-modified mRNAs.

**Figure 6:**
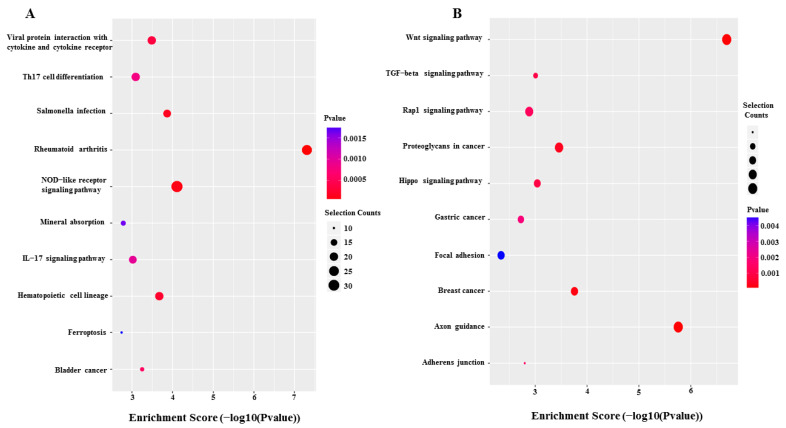
Kyoto Encyclopedia of Genes and Genomes (KEGG) pathway analysis of hypermethylated (**A**) and hypomethylated mRNAs (**B**).

GO and KEGG pathway analyses were performed to further explore the potential pathological and physiological processes in which differential m^6^A-tagged mRNAs are involved. GO analysis showed that these differentially m^6^A-modified mRNAs were involved in anatomical structure morphogenesis, anatomical structure development and developmental processes in the biological process module. They were involved in the plasma membrane, vesicles and cytoplasm in the cellular component module. Additionally, they were involved in protein binding, enzyme binding, chemokine activity and chemokine receptor binding in the molecular function module (Figure 5). KEGG pathway analysis showed that hypermethylated mRNAs were significantly enriched in viral protein interaction with cytokine and cytokine receptors (20 genes), Th17 cell differentiation (20 genes), *Salmonella* infection (18 genes) and the NOD-like receptor signaling pathway (32 genes), while hypomethylated mRNAs were significantly enriched in the Wnt signaling pathway (22 genes), TGF-beta signaling pathway (11 genes) and Rap1 signaling pathway (18 genes) (Figure 6).

## 4. Discussion

RNA methylation modification accounts for more than 60% of RNA chemical modifications, among which N6-methyladenine (m^6^A) is the most universal modification. The m^6^A modification exists widely in prokaryotes and eukaryotes and plays an important role in gene expression, RNA splicing, mRNA translation etc. Recently, the role of m^6^A modification in immune regulation and anti-infection has been revealed. For immune regulation, m^6^A methylation modification can regulate the function of dendritic cells (DC) and regulatory T cells. Decreased m^6^A levels lead to enhancement of suppressor of cytokine signaling (SOCS) RNA stability, thus inhibiting the IL2–STAT5 pathway, which has an important role in the suppressive function of Tregs [8]. Furthermore, studies by Lichinchi and Feng et al. demonstrated that m^6^A modification also plays a role in infection [15,16,18,19].

PJI (*S. aureus* is one of the main pathogens and is challenging to treat) is a catastrophic complication after joint arthroplasties. Multiple revision surgeries and antibiotic treatments are warranted to get effective treatment. However, even after two-stage revision and antibiotic treatment, about 4–17% of patients underwent recurrent infection [3], resulting in amputation or even death. With an increase of the number of joint arthroplasties, the number of recurrent infections is also increasing gradually, which brings physical pain to patients and economic burden to society [20]. It is therefore important that the pathophysiological process of PJI is comprehensively and thoroughly understood so as to improve the curative effect of PJI. Thus, this study was performed to more deeply understand the pathophysiological process of PJI.

To the best of our knowledge, this was the first study to explore m^6^A modification in PJI. The present work showed that the overall m^6^A level in the PJI group was significantly higher than that in the AF group. After screening for RNA methylases and demethylases, we found that there was a difference in the expression of METTL3 between the two groups (the PJI group was higher than the AF group). In order to clarify the RNA spectrum of differential methylation, we detected the differential m^6^A-methylated RNAs and found that there were 1834 hypermethylated mRNAs and 968 hypomethylated mRNAs. 

Bioinformatics analyses (GO and KEGG analyses for this study) were performed to thoroughly understand pathophysiological processes of PJI in which differential m^6^A-modified mRNAs are involved. The results showed that differential m^6^A-tagged mRNAs might have a role in following processes. 1. Infection and immune response: *S. aureus*, with various pathogen-associated molecular patterns (PAMPs), can be recognized by pattern recognition receptors (PRRs) (such as NOD-like receptors) that activate host immune and inflammatory responses [21]. The massive production of TGF-β can promote the differentiation and maturation of TH17 cells [22], which mainly participate in the process of pathogen clearance by secreting IL-17 [23]. In addition, *S. aureus* was previously considered to be an extracellular pathogen, but recent studies have demonstrated that it can also invade non-professional phagocytes such as osteoblasts, osteoclasts and osteocytes, causing intracellular infections similar to *Salmonella* [24,25,26]. m^6^A methylation modification might be involved in *S. aureus* infection (including intracellular infection) and host immune defense responses in PJI (as shown in the KEGG pathway analysis: *Salmonella* infection, NOD-like receptor signaling pathway, Th17 cell differentiation and IL-17 signaling pathway). 2. Bone metabolism: it has been shown that the Wnt signaling pathway has an important role in bone development and remodeling and is a critical mechanism for stimulating osteoblast differentiation and activity [27]. Rap1 is also involved in bone metabolism regulation by regulating osteoblast differentiation via an ERK/p38-mediated signaling pathway [28], and the Hippo signaling pathway has a role in regulating osteoclast formation [29]. Previous studies have shown that *S. aureus* infection could also lead to bone loss [30], but the mechanisms remain unclear. The present work demonstrated that m^6^A modification might be involved in regulation of bone loss in PJI mediated by Wnt, Rapa1 and Hippo signaling pathways (as shown in the KEGG pathway analysis: Wnt signaling pathway, Rap1 signaling pathway, Hippo signaling pathway and mineral absorption). 3. Programmed cell death: being stimulated by bacteria, PRRs (such as NOD-like receptor 3,4 etc.) bind with adaptor protein apoptosis-associated speck-like protein containing a CARD (ASC) to activate caspase-1. After that, activated caspase-1 shears Gasdermin D to activate pyroptosis [31]. At the same time, activated caspase-1 cleaves IL-1β and IL-18 precursors, which are then secreted out of cells to recruit inflammatory cells [31]. Ferroptosis, which was illustrated by Dixon et al. in 2012, is a new kind of programmed cell death that is quite different from apoptosis, necrosis and autophagy [32]. There were several studies indicating that both pyroptosis and ferroptosis play a key role in infection [33,34,35]. This study showed that m^6^A modification might be involved in this programmed cell death in PJI (as shown in the KEGG pathway analysis: NOD-like receptor signaling pathway and ferroptosis). Thus, therapeutic strategies targeted to m^6^A modification could be developed to regulate NOD-like receptor signaling pathways and ferroptosis, both of which play an important role in host’s resistance to infection. This could enhance its anti-infection roles, which should be clarified in further research and may serve as a potential target for developing effective treatment strategies.

The biggest limitation of this study is that these pathways were obtained by bioinformatics analysis. Thus, further functional verification is warranted. Moreover, the number of PJI cases included in this study was relatively small, so the results should be interpreted with caution.

## 5. Conclusions

In summary, this study showed that there was a difference in the overall m^6^A level between PJI and AF patients, and m^6^A modification might be involved in the regulation of a variety of pathophysiological processes in PJI. The present work broadened our understanding of the pathophysiological process of PJI and lays a foundation for the development of more effective treatment strategies.

## Data Availability

Data will be made available on request.

## Supplementary Materials

The following supporting information can be downloaded at: https://www.mdpi.com/article/10.3390/jcm12082863/s1, File S1: Primers used for qPCR in this study; File S2: Demographic characteristics; File S3: Top 20 differential m6A-modification mRNAs.
