# Supplementary material for: Alteration of m6A-Tagged RNA Profiles in Bone Originated from Periprosthetic Joint Infection
Source: J Clin Med. 2023 Apr 14;12(8):2863. doi: 10.3390/jcm12082863 (PMC10146075; doi:10.3390/jcm12082863)
Supplement: Supplementary file 1 [file jcm-12-02863-s001.zip › File S1.pdf]

Supplementary File S1: Primers used for qPCR in this study.

Primer	Forward	Reverse
GAPDH	GGAGCGAGATCCCTCCAAAAT	GGCTGTTGTCATACTTCTCATGG
METTL3	TTGTCTCCAACCTTCCGTAGT	CCAGATCAGAGAGGTGGTGTAG
METTL14	AGTGCCGACAGCATTGGTG	GGAGCAGAGGTATCATAGGAAGC
WATP	CTTCCCAAGAAGGTTCGATTGA	TCAGACTCTCTTAGGCCAGTTAC
FTO	AACACCAGGCTCTTTACGGTC	TGTCCGTTGTAGGATGAACCC
ALKBH5	CGGCGAAGGCTACACTTACG	CCACCAGCTTTTGGATCACCA
