# Supplementary material for: Alteration of m6A-Tagged RNA Profiles in Bone Originated from Periprosthetic Joint Infection
Source: J Clin Med. 2023 Apr 14;12(8):2863. doi: 10.3390/jcm12082863 (PMC10146075; doi:10.3390/jcm12082863)
Supplement: Supplementary file 1 [file jcm-12-02863-s001.zip › File S2.pdf]

Supplementary file S2: Demographic characteristics.

ID	Sex	Age	BMI	Joints	Diagnosis	Pathogen	Charlson comorbidity index
1	Female	60	24.3	Hip	PJI	*S. aureus*	2
2	Female	56	23.6	Hip	PJI	*S. aureus*	2
3	Female	63	22.4	Hip	PJI	*S. aureus*	3
4	Female	57	23.6	Hip	AF	-	3
5	Female	58	22.8	Hip	AF	-	2
6	Female	63	23.7	Hip	AF	-	3

PJI: prosthetic joint infection; AF: aseptic failure; *S. aureus*: *Staphylococcus aureus.*
