# Supplementary material for: Alteration of m6A-Tagged RNA Profiles in Bone Originated from Periprosthetic Joint Infection
Source: J Clin Med. 2023 Apr 14;12(8):2863. doi: 10.3390/jcm12082863 (PMC10146075; doi:10.3390/jcm12082863)
Supplement: Supplementary file 1 [file jcm-12-02863-s001.zip › File S3.pdf]

Supplementary File S3: Top 20 differential m⁶A-modification mRNAs.

Gene Symbol	Foldchange(log2-scaled)	Regulation	RNA length	Chromosome	Strand	*P*-value
CSF2	8.586921172	hyper	787	chr5	+	0.000309929
HLA-DRB5	8.302686099	hyper	1260	chr6	-	0.000201517
SAA2	6.882009984	hyper	563	chr11	-	0.0081095
CATG00000054068.1	6.458969291	hyper	697	chr20	-	0.005136763
C15orf48	6.119966081	hyper	875	chr15	+	0.003076867
IL1A	6.006090494	hyper	2124	chr2	-	0.004085226
CXCL6	5.892684401	hyper	1718	chr4	+	7.54543E-06
MMP3	5.822165409	hyper	2016	chr11	-	0.018529237
OSM	5.7982906	hyper	1854	chr22	-	0.000588982
MARCO	5.63937407	hyper	1838	chr2	+	0.001124623
HBG2	-6.780830576	hypo	614	chr11	-	0.013155041
CTD-2643I7.4	-5.563853553	hypo	651	chr11	-	0.012842058
CATG00000107414.1	-5.194026574	hypo	2974	chr9	+	0.011114276
OMD	-5.167011575	hypo	2449	chr9	-	0.03754457
FABP4	-5.099003774	hypo	941	chr8	-	0.026677007
EPHA3	-5.07345817	hypo	5809	chr3	+	0.021092687
MYRIP	-4.991866646	hypo	5077	chr3	+	0.016251428
IFIT1B	-4.977426788	hypo	1972	chr10	+	0.037797445
ADGRG6	-4.791112759	hypo	7026	chr6	+	0.010185306
TMEM132C	-4.750943442	hypo	4947	chr12	+	0.024285092
